# Atypical Nodular Pulmonary Kappa Light-Chain Deposition

**DOI:** 10.1155/2021/5578885

**Published:** 2021-03-10

**Authors:** Jessy Nellipudi, John Brealey, Sonja Klebe, David Lance

**Affiliations:** ^1^Department of Cardiothoracic Surgery, Flinders Medical Centre, Southern Adelaide Local Health Network, South Australia, Australia; ^2^College of Medicine and Public Health, Flinders University, South Australia, Australia; ^3^Department of Anatomical Pathology, Flinders Medical Centre, Southern Adelaide Local Health Network, South Australia, Australia; ^4^Fellow of the Royal Australian College of Surgeons, Australia

## Abstract

We report a case of an incidental positron emission tomography avid right middle lobe lesion which was increasing in size. Due to concerns regarding malignancy, the patient underwent right middle lobectomy. Microscopic examination showed a 12 × 10 × 10 mm poorly circumscribed lesion composed of eosinophilic material. The material labelled strongly for kappa light chains; however, Congo red stain was only weakly positive and without “apple-green” positive birefringence under polarised light. Electron microscopy revealed fibrillar amyloid-like material. The features were those of kappa light-chain deposition.

## 1. Introduction

Amyloid is a common manifestation of tissue deposition of abnormal proteins. It is defined as extracellular deposition of fibrillar proteinaceous material that stains positively with Congo red stain and shows apple-green birefringence under polarised light, with beta-pleated sheet structure by electron microscopy (EM) [1]. Localised nodular amyloid deposition in the lung (pulmonary amyloidoma) is rare and usually found incidentally on chest radiography in older adults [2]. Kappa light chains are a common cause of amyloid; in some instances, kappa light chains form deposits distinct from amyloid. This is referred to as light-chain deposition disease [3], very rarely manifesting within the lung, where it may be seen in diffuse or nodular forms [4]. We report a case of pulmonary kappa light-chain deposition in a 67-year-old Caucasian man.

## 2. Case Report

**Figure 1:**
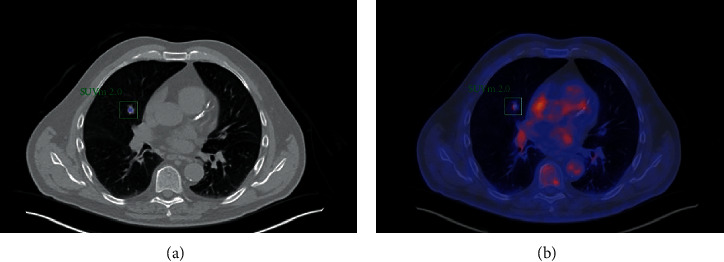
(a) Computed tomography image with the corresponding (b) positron emission tomography image showing 16 mm right middle lobe lung lesion (FDG avid, SUV max 2.0).

A 67-year-old male ex-smoker (>20 years) with a 30 pack-year history was investigated for morning cough and clear sputum with no constitutional symptoms and an active lifestyle. Past medical history included chronic obstructive pulmonary disease and ischaemic heart disease. External physical examination was unremarkable. Chest computed tomography (CT) showed a right middle lobe nodule which increased from 11 mm to 16 mm over six months. A positron emission tomography (PET) scan showed low radiolabeled [18F]-2-fluoro-2-deoxy-D-glucose (FDG) uptake with a standardised uptake value (SUV) max of 2.0 and no evidence of metastatic disease (Figure 1). CT-guided biopsy identified eosinophilic amorphic material, which was Congo red negative, with no definitive diagnosis reached. Due to the volume increase of the suspicious lesion, a right middle lobe lobectomy was performed.

## 3. Microscopy

**Figure 2:**
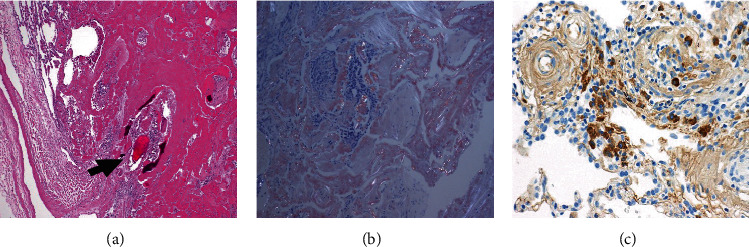
(a) Dense extracellular eosinophilic material, presenting as a mass-like deposit with areas of calcification and ossification (arrow), perivascular eosinophilic deposits were noted, especially at the periphery of the mass-like deposit, and are seen better in (c). Perivascular eosinophilic material was not seen diffusely throughout the lung (magnification ×400). (b) Congo red staining when viewed under polarised light (apple-green birefringence under polarised light not visualised) (magnification ×400). (c) Kappa light-chain immunochemistry shows positivity within the amorphous material and plasma cells (^) (magnification ×400).

Microscopic examination showed a 12 × 10 × 10 mm poorly circumscribed lesion composed of dense eosinophilic material with areas of calcification and ossification. Perivascular eosinophilic material was noted, especially at the periphery of the mass-like deposit, and was not seen diffusely throughout the lung (Figure 2(a)). Congo red stain was only weakly positive and did not demonstrate classical “apple-green” positive birefringence under polarised light (Figure 2(b)). The material labelled strongly for kappa (k), but not for lambda (*λ*) light chains, nor amyloid P. A moderate number of plasma cells showed labelling exclusively for k light chains, raising the possibility of a monoclonal population (Figure 2(c)). The labelling for k light chains highlighted plasma cells. The surrounding lung parenchyma showed no significant pathology.

## 4. Electron Microscopy

**Figure 3:**
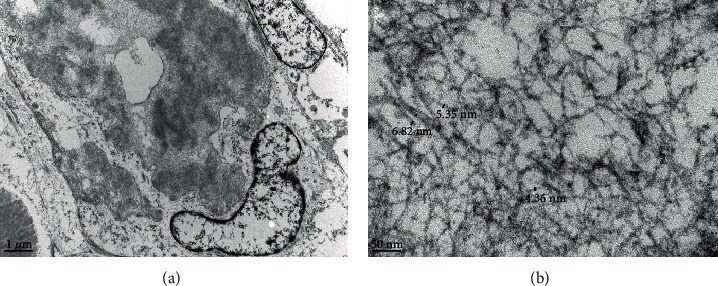
Electron microscopy: (a) electron-dense amyloid-like material (scale bar = 1 micron); (b) fibrils measuring 4-7 nm in diameter observed at higher magnification (scale bar = 50 nanometer).

The material in question was scattered throughout the lung parenchyma and occasionally was adjacent to plasma cells. At low magnification, the material had a worm-like appearance measuring 10-20 microns in diameter. In cross-section, the material was composed of a central core of electron-dense material that ultrastructurally resembled elastin and an outer zone of amyloid-like fibrils (not shown in the figure). In one area, fibrils were more dispersed and measured 4-7 nm in diameter (Figure 3).

## 5. Haematology

Haematology workup showed no serum paraproteins nor evidence of B cell monoclonality, no atypical expression of T cell markers. The serum kappa/lambda light-chain ratio was 1.4 (reference range: 0.25–1.65). There were no urinary Bence Jones proteins and no bony lytic lesions on whole-body CT. Bone marrow biopsy was not performed.

## 6. Comment

Immunoglobulin light-chain deposits in tissues can present as primary amyloid light-chain (AL) amyloidosis (AL amyloidosis) or light-chain deposition disease (LCDD) [5]. AL amyloidosis is a monoclonal plasma cell or rarely lymphoplasmacytic proliferative disorder in which monoclonal immunoglobulin light chains are deposited throughout tissues. AL amyloid deposits are composed of protein fibrils with a common core structure, consisting of anti-parallel *β*-strands (less commonly, parallel *β*-strands). This specific, highly ordered ultrastructure of amyloid fibrils accounts for their characteristic property of binding Congo red dye when viewed under cross-polarised light produces the classical “apple-green” birefringence [1, 5]. Similar to AL amyloidosis, LCDD may be related to monoclonal plasma cell proliferative disorder, or it may rarely be idiopathic (ICD-O-coded 9769/1) [3] and can involve the lung as a diffuse or a nodular form. Pulmonary light-chain deposition (PLCDD) is rare, especially in the localised form. Similar to AL amyloidosis, PLCDD also demonstrates positive k light-chain immunochemistry within amorphous material [6]. However, in PLCDD, light chains failed to assume the *β*-pleated sheet configuration and result in granular deposits seen by electron microscopy (EM) and lack the disordered meshwork of 8-10 nm fibrils, characteristics of amyloid [5–7]. Consequently, PLCDD can be differentiated from amyloid by the absence of Congo red staining and distinct EM appearances [6, 7].

In the World Health Organisation (WHO) 2017 revised classification of tumours of haematopoietic and lymphoid tissue, LCDD is classified under the monoclonal immunoglobulin disposition diseases. Whilst LCDD frequently occur in association with plasma cell myeloma or in patients with M protein and marrow plasma cells as monoclonal gammopathy of undetermined significance, some cases are idiopathic or occur in association with another lymphoproliferative disorder [3]. Given the morphological and immunochemical characteristics of the lesion in question, in this case, a firm diagnosis is unable to be established, and morphological may represent an intermediate between AL amyloidosis and PLCDD.

Our patient presented with a solitary FDG avid (SUV max 2.0) nodule, which was concerning for cancer. Hence, the decision to proceed with lobectomy was made with diagnostic and curative intent. Microscopically, the material was densely eosinophilic and on Hematoxylin and Eosin (H&E) staining suggestive of amyloid. However, Congo red staining did not reveal the classical appearance of “apple-green” birefringence and amyloid P was negative. The material labelled strongly for k light chain but not for *λ*-light chain, and electron microscopy revealed fibrillar amyloid-like material measuring 4-7 nm and larger worm-like formations 10-20 microns in diameter. The material in question was unusual in that it had an amyloid-like component. However, the diameter of most of the fibrillar substructure is less than that observed for amyloid (typically 7-12 nm).

Congo red staining is a screening test, but mass spectroscopy in conjunction with immunohistochemistry is now regarded as the “gold standard” for the diagnosis of amyloid since heterogeneity in Congo red staining has been reported [8]. Electron microscopy is more objective as fibril diameter can be measured and in this case was clearly less than that typical of amyloid, further supported by the lack of Congo red staining and labelling for P-protein. The difficulty in this case, which showed focal amyloid-like features on EM, is how to best designate the lesion. Kappa light-chain deposition with focal amyloidoma-like features, as an intermediate between focal amyloid and PLCDD, may be deemed the best designation.

PET scan is an important diagnostic and staging modality for the evaluation of pulmonary nodules. Concerning PET scan in the assessment of pulmonary amyloid lesions, there is varying evidence of pulmonary amyloid lesions showing intense to no FDG uptake [9]. Light-chain deposition disease has also been reported to show varying degree of FDG uptake [7]. Whilst PET scans are important in evaluating lung lesions, their use in the detection of pulmonary amyloid or light-chain deposition is not well established.

Furthermore, pulmonary light-chain deposition may also be associated with underlying lymphoproliferative or immunological disorders. In a case series of 46 patients conducted by Milani et al. [10], 11 patients had urinary or serum monoclonal proteins detected, 13 patients had abnormal free chain ratio, Sjogren's disease was diagnosed in three patients, and MALT cell lymphoma was diagnosed in two patients [10]. Therefore, further haematological workup is essential. This case did not reveal any suggestion of systemic amyloidosis, multiple myeloma, or evidence of other lymphoproliferative disorders, therefore necessitating clinical surveillance.

In conclusion, we describe a case of k light-chain deposition whose morphological, histochemical, and electron microscopy features do not allow classification within a specific category recognised by the current WHO classification, thus expanding the spectrum of monoclonal immunoglobulin deposition disease lesions. Regardless of the diagnosis, haematological review to identify any underlying lymphoproliferative diseases is essential since they may present with hematolymphoid malignancies after prolonged follow-up.

## References

[1] Wechalekar A. D., Gillmore J. D., Hawkins P. N. (2016). Systemic amyloidosis. *The Lancet*.

[2] Matsumoto K., Ueno M., Matsuo Y., Kudo S., Horita K., Sakao Y. (1997). Primary solitary amyloidoma of the lung: findings on CT and MRI. *European Radiology*.

[3] CE S. S. H., Harris N. L., Jaffe E. S., Pileri S. A., Stein H., Thiele J. (2017). WHO Classification of Tumours of Haematopoietic and Lymphoid Tissues.

[4] Yee M., Delahunt B., Russell P. A. (2016). Nodular pulmonary light chain deposition disease. *Pathology*.

[5] Bhargava P., Rushin J. M., Rusnock E. J. (2007). Pulmonary light chain deposition disease: report of five cases and review of the literature. *American Journal of Surgical Pathology*.

[6] Wei P., Tao R., Liu Y. (2020). Pulmonary light chain deposition disease: a case series and literature review. *Annals of translational medicine*.

[7] Baqir M., Moua T., White D., Yi E. S., Ryu J. H. (2020). Pulmonary nodular and cystic light chain deposition disease: a retrospective review of 10 cases. *Respiratory Medicine*.

[8] Bowen K., Shah N., Lewin M. (2012). AL-amyloidosis presenting with negative Congo red staining in the setting of high clinical suspicion: a case report. *Case reports in nephrology*.

[9] Dong M.-J., Zhao K., Liu Z.-F., Wang G.-L., Yang J. (2015). Primary pulmonary amyloidosis misdiagnosed as malignancy on dual-time-point fluoro-deoxyglucose positron emission tomography/computed tomography: a case report and review of the literature. *Oncology Letters*.

[10] Milani P., Basset M., Russo F., Foli A., Palladini G., Merlini G. (2017). The lung in amyloidosis. *European Respiratory Review*.

